# THE INNER DYNAMICS OF THE OLD AND SOCIALLY WITHDRAWN IN JAPAN: INSIGHTS FOR INTERVENTIONS FROM CASE STUDY ANALYSES

**DOI:** 10.1093/geroni/igae098.2547

**Published:** 2024-12-31

**Authors:** Tomoko Ikeuchi, Cullen Hayashida

**Affiliations:** Tokyo Metropolitan Institute for Geriatrics and Gerontology, Tokyo, Japan; University of Hawaii, Honolulu, Hawaii, United States

## Abstract

An increasing number of older adults in Japan are refusing social support even when offered. This lack of support elevates their risk of health deterioration, delays evacuation in disaster scenarios, and increases the likelihood of premature and lonely deaths with inadequate closures. This study examines the psychological factors contributing to the lack of motivation among socially isolated older adults in Japan to seek help. We conducted semi-structured one-on-one interviews with nine individuals aged 60 years or older who reside in the community identified as socially isolated by community-based healthcare personnel. Using a narrative case study approach, the study revealed a prevalent concern among these socially isolated individuals about causing inconvenience to others by seeking help. The findings suggest that their reluctance to ask for help may be driven not by a desire to maintain independence but rather by a concern about inconveniencing others. In the context of Japanese culture, maintaining harmony with others is considered important. Asking for help might inconvenience others and potentially disrupt this harmony. To effectively assist those who refuse necessary support, social and community health workers should develop ways of minimizing the perceived burden of the recipients. However, further research is necessary to establish whether these findings are unique to the Japanese cultural context. Examples of innovative strategies to address this challenge will be discussed.

